# Toxic Epidermal Necrolysis Superimposed on Severe Drug Rash With Eosinophilia and Systemic Symptoms Complicated by Fatal Hemophagocytic Lymphohistiocytosis: A Case Report

**DOI:** 10.7759/cureus.69503

**Published:** 2024-09-16

**Authors:** Gowri Swaminathan, Daniel Miller, Nicole Noff, Zara Bhutta, Jonathan Muratori, Faateh Rauf, Santino Patrizi, Bike Ozkan, Ricardo Lopez

**Affiliations:** 1 Internal Medicine, Icahn School of Medicine at Mount Sinai/NYC Health and Hospitals/Queens Hospital Center, New York, USA

**Keywords:** critical care medicine, drug reaction with eosinophilia and systemic symptoms, hemophagocytic syndrome, primary hemophagocytic lymphohistiocytosis, toxic epidermal necrolysis

## Abstract

Drug rash with eosinophilia and systemic symptoms (DRESS) is a severe cutaneous adverse reaction (SCAR) characterized by an extensive skin rash associated with visceral organ involvement, fever, eosinophilia, atypical lymphocytosis, and lymphadenopathy. Toxic epidermal necrolysis (TEN) is a more severe, distinct adverse cutaneous reaction that causes extensive necrosis and detachment of the epidermis, involving over 30% of the body surface area (BSA). Hemophagocytic lymphohistiocytosis (HLH), a form of excessive immune activation, is known to be associated with SCARs such as DRESS. We present a peculiar case of overlap between different SCARs to reiterate their gravity, a severe form of DRESS triggered by the use of allopurinol overlapping with the aggressive TEN possibly from interaction with vancomycin administered for severe sepsis, which was complicated by a fatal case of HLH.

## Introduction

Severe cutaneous adverse reactions (SCARs) to drugs include acute generalized exanthematous pustulosis (AGEP), drug reaction with eosinophilia and systemic symptoms (DRESS), and epidermal necrolysis (Steven-Johnson syndrome-toxic epidermal necrolysis (SJS-TEN)). Overlapping SCARs are categorized as cases fulfilling the criteria for definite or possible diagnosis of at least two adverse drug reactions (ADRs) mentioned above [1]. It is pertinent that the culprit drug be identified to avoid further exposure, as the timeline can eventually determine the course of the overlapping conditions and patient outcomes.

Drug-specific T-cell lymphocytes are a result of cross-reactivity with already sensitized effector memory T-cell lymphocytes after infection, and an exaggerated immune response can occur after a viral reactivation or administration of an incriminating drug with or without concomitant infection [2]. The diagnostic criteria for hemophagocytic lymphohistiocytosis (HLH) can either be molecular with elevated inflammatory markers or clinical, where five of the following nine findings are evident: (1) fever ≥ 38.5°C; (2) splenomegaly; (3) peripheral blood cytopenia, with at least two of the following: hemoglobin < 9 g/dL, platelets < 100,000/microL, and absolute neutrophil count < 1,000/microL; (4) hypertriglyceridemia and/or hypofibrinogenemia (fibrinogen < 150 mg/dL); (5) hemophagocytosis in bone marrow, spleen, lymph node, or liver; (6) low or absent NK cell activity; (7) ferritin > 500 ng/mL; (8) elevated soluble CD25; and (9) elevated CXCL9 [3]. Our case report underscores the poor prognosis when SCARs overlap in their most severe forms, i.e., overlapping severe DRESS and TEN in our patient, complicated by a life-threatening case of HLH due to exaggerated immune activation. This case report is unique, as such a devastating overlap of these conditions has not been previously reported.

## Case presentation

A 54-year-old male presented to the emergency department (ED) with high fevers of five days duration. The patient had recently been treated for emphysematous pyelonephritis in the setting of a urinary tract infection (UTI) secondary to extended-spectrum beta-lactamase-producing *Escherichia coli.* The patient had received piperacillin-tazobactam through a peripherally inserted central catheter (PICC), which was removed five days before the current encounter. He had a history of prior pelvic trauma and had undergone multiple genitourinary surgeries. The patient also had a significant past medical history of three-vessel coronary artery disease, status-post coronary stents, hypertension, hyperlipidemia, and chronic kidney disease (CKD) V.

**Figure 1:**
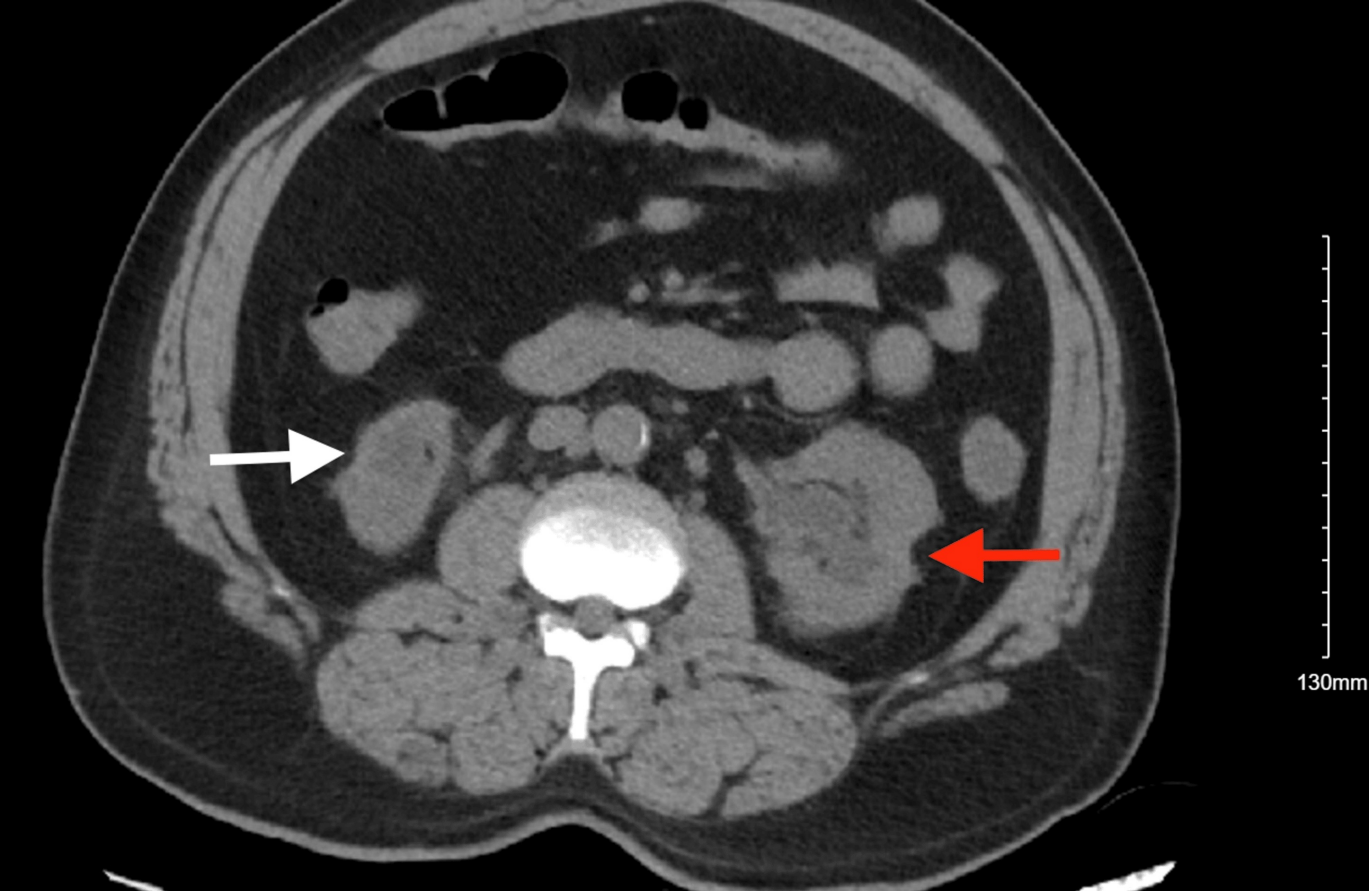
CT of the abdomen and pelvis (axial view) without contrast showing atrophic right kidney (white arrow) and left hydronephrosis (red arrow) with bilateral cortical scarring CT: computed tomography

**Figure 2:**
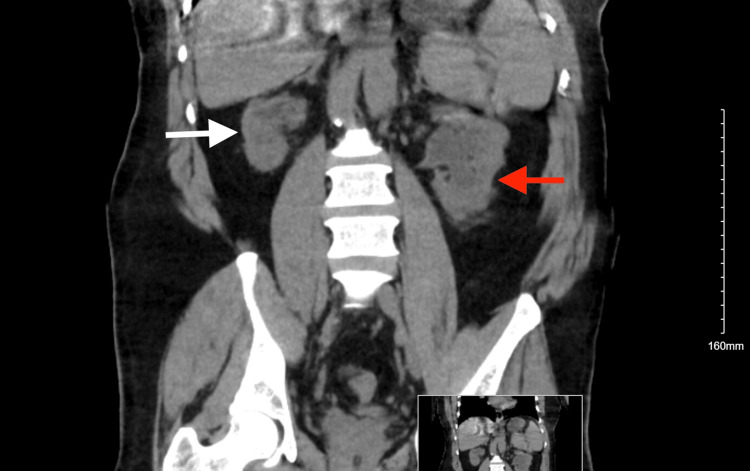
CT of the abdomen and pelvis (coronal view) without contrast showing atrophic right kidney (white arrow) and left hydronephrosis (red arrow) with bilateral cortical scarring CT: computed tomography

Upon presentation, he was ill-appearing, febrile to 102.1°F, tachycardic to 116 beats/minute, tachypneic to 30 breaths/minute, and hypotensive to 84/70 mmHg. The patient was found to have bilateral crackles upon auscultation. His serum bicarbonate levels were 14 mmol/L, and creatinine was 10.79 mg/dL (baseline was about 6 mg/dL one month before); the patient was also found to be COVID-19-positive. He was given a dose of vancomycin and piperacillin-tazobactam in the ED and was admitted to the intensive care unit (ICU) in the setting of impending decompensation. Computed tomography (CT) of the abdomen and pelvis revealed unchanged left kidney hydronephrosis and right kidney atrophy from prior (Figure 1 and Figure 2). Laboratory tests were suggestive of acute kidney injury (AKI) superimposed on CKD V (Table 1). The patient was started on emergent hemodialysis and was also started on sodium bicarbonate infusion as a temporizing measure. The patient remained tachypneic despite a trial of bi-level positive airway pressure (BiPAP) and bicarbonate supplementation. He was intubated and placed on mechanical ventilation. Shortly after intubation, the patient sustained a cardiac arrest with a return of spontaneous circulation in four minutes after advanced cardiac life support (ACLS) was activated. He was started on vasopressors.

**Table 1:** Laboratory values reflecting the patient's worsening renal function (AKI on CKD V upon admission) AKI: acute kidney injury, CKD: chronic kidney disease, BUN: blood urea nitrogen, GFR: glomerular filtration rate

	Normal range	November 2022	June 2023	Late July 2023	Early August 2023	August 2023 (admission)
BUN	6-23 mg/dL	57 mg/dL	67 mg/dL	68 mg/dL	49 mg/dL	88 mg/dL
Creatinine	0.70-1.20 mg/dL	4.21 mg/dL	5.70 mg/dL	6.70 mg/dL	6.20 mg/dL	12.47 mg/dL
GFR	≥60 mL/minute/m^2^	16 mL/minute/m^2^	11 mL/minute/m^2^	9 mL/minute/m^2^	10 mL/minute/m^2^	4 mL/minute/m^2^
Serum bicarbonate	22-29 mmol/L	20 mmol/L	22 mmol/L	20 mmol/L	22 mmol/L	14 mmol/L

**Figure 3:**
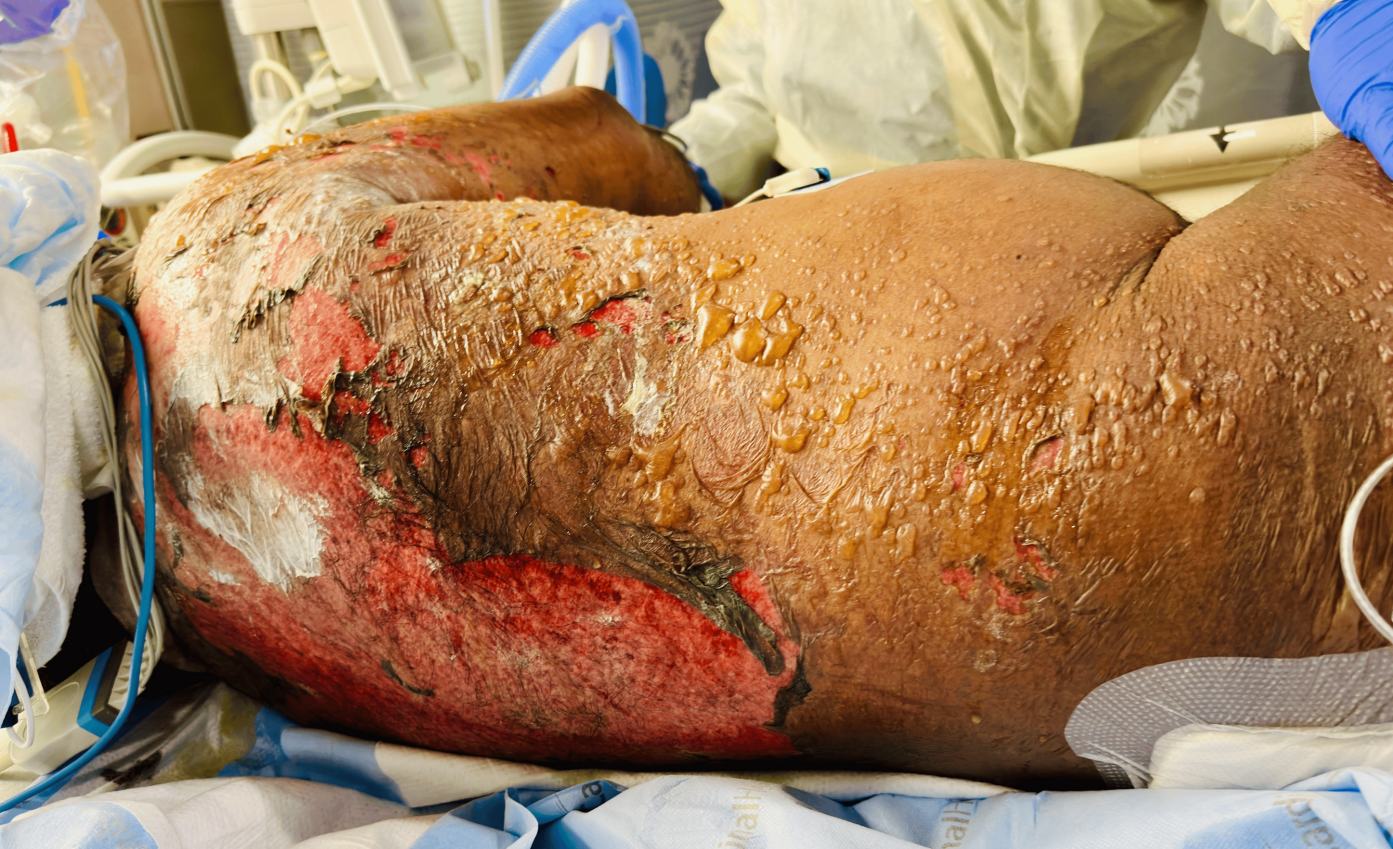
Extensive desquamating lesions of toxic epidermal necrolysis on day 3 covering more than 30% of the body surface area

The patient had been started on remdesivir initially; however, the medication was discontinued as the clinical picture was strongly suggestive of sepsis due to UTI/pyelonephritis rather than COVID-19 itself. Sputum culture returned positive for *Burkholderia multivorans*, sensitive to meropenem, and the antibiotics were switched accordingly. He continued to have intermittent high-grade fevers, and his vasopressor requirement continued to climb. At the start of day 2, he was already on three vasopressors. He started to develop skin-colored bullae over his upper back and neck on the background of mottled skin, which were not discernible before. They blistered quickly, oozing serous fluid. He was additionally started on clindamycin and cefazolin. The bullae progressed to involve more than 30% of the skin (Figure 3), with a positive Nikolsky sign. There was high suspicion for TEN, but the transfer to a burn center was deferred due to hemodynamic instability. A review of the patient's electronic medical record (EMR) showed that he had been started on allopurinol for gout about five weeks back; however, it was not resumed during his current hospitalization. His recent laboratory results showed that he had been having progressive peripheral blood eosinophilia along with elevated creatine phosphokinase (CPK). This pointed toward the diagnosis of severe DRESS with a RegiSCAR (registry of SCAR scoring system) score of 6, which indicates a definite case of DRESS (Table 2), in the background of peripheral blood eosinophilia > 1.5×10^9^/L, rash involving >50% of the body surface area, and multiorgan involvement [4]. He was started on continuous renal replacement therapy (CRRT). He began showing features of disseminated intravascular coagulation and was transfused with blood products. He continued to spike high temperatures of 105.5°F, unresponsive to therapies.

**Table 2:** RegiSCAR scoring system for diagnosing DRESS The diagnosis of DRESS syndrome is based on the total score: <2 points: no case; 2-3 points: possible case; 4-5 points: probable case; and >5 points: definite case [4]. Our patient had a RegiSCAR score of 6: 2 for peripheral blood eosinophilia of ≥1.5×10^9^/L; 2 for skin rash, suggesting DRESS with an extent of >50% of the BSA; and 2 for organ involvement, indicating definite DRESS. ANA: antinuclear antibody, BSA: body surface area, DRESS: drug rash with eosinophilia and systemic symptoms, HAV: hepatitis A virus, HBV: hepatitis B virus, HCV: hepatitis C virus, N: no, U: unknown, WBC: white blood cell, Y: yes

Items	Score			Comments
	-1	0	1	
Fever ≥ 38.5°C	N/U	Y	-	-
Enlarged lymph nodes	-	N/U	Y	>1 cm and ≥2 different areas
Eosinophilia ≥ 0.7×10^9^/L or ≥10% if WBC < 4×10^9^/L	-	N/U	Y	Score 2, when ≥ 1.5×10^9^/L or ≥ 20% if WBC < 4×10^9^/L
Atypical lymphocytosis	-	N/U	Y	-
Skin rash	-			Rash suggesting DRESS: ≥ 2 symptoms: purpuric lesions (other than legs), infiltration, facial edema, psoriasiform desquamation
Extent > 50% of the BSA	-	N/U	Y	
Rash suggesting DRESS	N	U	Y	
Skin biopsy suggesting DRESS	N	Y/U	-	-
Organ involvement	-	N	Y	Score 1 for each organ involvement, maximal score: 2
Rash resolution ≥ 15 days	N/U	Y	-	-
Excluding other causes	-	N/U	Y	Score 1 if 3 tests of the following tests were performed and all were negative: HAV, HBV, HCV, mycoplasma, chlamydia, ANA, and blood culture

A point-of-care ultrasound examination of the heart revealed a low preload due to intravascular volume depletion despite aggressive fluid resuscitation and a reduced ejection fraction due to a history of ischemic cardiomyopathy; however, no formal echocardiography was found on file. He was given several boluses of plasmalyte and was started on a fourth vasopressor, phenylephrine. CPK levels continue to climb up to 18,120 U/L, indicating severe rhabdomyolysis. The patient was not able to tolerate further sessions of CRRT. He had elevated ferritin levels of >10,000 ng/mL and soluble IL-2 receptor alpha (sCD25) at 63,180 pg/mL, along with high-grade fever, pancytopenia, deranged liver function tests, and hypofibrinogenemia, pointing toward the diagnosis of hemophagocytic lymphohistiocytosis (HLH). Bone marrow biopsy was deferred due to hemodynamic instability. At the end of day 3, the patient died after a cardiopulmonary arrest.

## Discussion

Drug-induced severe cutaneous adverse reactions (SCARs) include AGEP, DRESS, SJS, and TEN or an overlap between the two (SJS-TEN). These conditions are distinct entities that come with individual clinical, biological, and histological patterns; however, it is imperative that a distinction be made early into the course of hospitalization, as it may dictate the clinical outcome [5]. True overlapping SCARs are extremely rare, making our case unique, with a true overlap of DRESS and TEN, complicated by fatal HLH.

DRESS is a drug reaction that occurs 1-8 weeks after the introduction of the causative drug. It starts as a pruriginous maculopapular rash over the face and spreads all over; the condition is frequently associated with edema of the face and extremities, fever, and peripheral lymphadenopathies. DRESS lesions have been reported to continue evolving despite the cessation of the offending drug, which was observed in our patient. Mucosal involvement is typically rare, but systemic involvement is characteristic of severe DRESS in the form of hepatic and renal impairment, as seen in this case. Laboratory changes are marked by peripheral blood eosinophilia, atypical lymphocytes, and possibly elevated liver enzymes and/or decreased glomerular filtration rate (GFR) [6]. The positive Nikolsky sign and the desquamative skin lesions involving >30% of the body surface area in our patient pointed to a more severe condition, TEN. The Severity-of-Illness Score for Toxic Epidermal Necrolysis (SCORTEN) (Table 3 and Table 4) for our patient was 6 (1 point each for age > 40 years, heart rate over 120 per minute, serum blood urea nitrogen > 28 mg/dL, >10% body surface area involved, serum bicarbonate < 20 mEq/L, and serum blood glucose of >250 mg/dL), indicating a mortality rate of >90% (Table 4) [7].

**Table 3:** Independent risk factors in SCORTEN and the scores assigned to each risk factor SCORTEN: Severity-of-Illness Score for Toxic Epidermal Necrolysis Source: [7]

Risk factor	Score	
-	0	1
Age	<40 years	≥40 years
Associated cancer	No	Yes
Heart rate	<120 beats/minute	≥120 beats/minute
Serum blood urea nitrogen	≤28 mg/dL (10 mmol/L)	>28 mg/dL (10 mmol/L)
Detached or compromised body surface	<10%	≥10%
Serum bicarbonate	≥20 mEq/L (≥20 mmol/L)	<20 mEq/L (<20 mol/L)
Serum glucose	≤250 mg/dL (≤13.88 mmol/L)	>250 mg/dL (>13.88 mmol/L)

**Table 4:** Mortality rates and relative risks according to the SCORTEN level Based on the development sample size of 165 patients in the study conducted by Fouchard et al. [7] SCORTEN: Severity-of-Illness Score for Toxic Epidermal Necrolysis, CI: confidence interval

SCORTEN	Mortality rate		Odds ratio (95% CI)
	Percent	95% CI	
0-1	3.2	0.1-16.7	1
2	12.1	5.4-22.5	4.1 (0.5-35.2)
3	35.3	19.8-53.5	14.6 (2.0-138.0)
4	58.3	36.6-77.9	42.0 (4.8-367.0)
≥5	90.0	55.5-99.8	270 (15.0-487.0)

The three entities (DRESS, SJS-TEN, and AGEP) are categorized as type IV delayed hypersensitivity reactions (according to the Coombs and Gell classification). These are mediated by T-cells, which can activate different chemokines and inflammatory cells, resulting in various clinical patterns based on the cells activated. Specific alleles of human leukocyte antigens (HLAs) are involved in the pathogenesis, leading to different activation pathways; however, a dominant pathway drives the pathogenesis [8]. Still, sometimes, these immune reactions can overlap and result in rare true overlaps such as our case, the incidence of which was only 2.1% among the 216 cases of SCARs, as reported by Bouvresse et al. upon applying the RegiSCAR validation scores [1].

Hemophagocytic disorders, which are severe conditions causing life-threatening immune activation, can be genetic in origin, i.e., primary hemophagocytic lymphohistiocytosis (HLH), or can result from infections, rheumatological diseases, immunodeficiency, or metabolic diseases, i.e., secondary HLH or macrophage activation syndrome (MAS). Cytopenias, a very high ferritin level of >10,000, and liver function derangement have been found to be very helpful in distinguishing HLH from other conditions; in fact, the frequency of liver function abnormalities in HLH is so high that the absence of the same should prompt one to search for alternate diagnoses [9]. Our patient fulfilled all three criteria, making a solid case for HLH. Jordan et al. found that sIL-2R/sCD25 correlates with disease activity the most, which was elevated in our patient [10]. The poor prognostic factors in TEN, such as pancytopenia and visceral organ involvement, as reported by Rajaratnam et al., suggest the presence of underdiagnosed HLH associated with TEN, as described in our case [11]. The mainstay of treatment for HLH has been described as steroids, and recently, other immunosuppressive therapies such as anakinra (an IL-1 blocker), ruxolitinib (JAK1/2 inhibitor), alemtuzumab (CD52 monoclonal antibody), and emapalumab (anti-IFN-γ monoclonal antibody) have been reported with some success. However, with or without treatment, the mortality rate in HLH is very high; in a national retrospective study conducted by Abdelhay et al., the in-hospital mortality rate was as high as 18.4%, and they did not find any significant change in rates of in-hospital mortality (slope = −0.01; p = 0.95) or administration of in-hospital HLH treatment (slope = 0.46, p = 0.20) [12]. Although our patient received steroids during his hospital course, he did not survive the overlapping SCARs in their most severe forms.

## Conclusions

HLH is an underdiagnosed condition, and the concept of overlapping SCARs continues to evolve, especially in the setting of newly introduced drugs and their interaction with time-tested drugs. In the presented case, allopurinol is the most probable agent responsible for triggering the SCARs, with a possible contribution from vancomycin. It is essential to clinch the diagnosis early to facilitate a favorable clinical outcome. We presented this complex case to illustrate the severity of the overlap of different drug-induced hypersensitivity reactions and how they can complicate a patient's recovery, proving to be fatal in rare scenarios. Our presented case, which started as sepsis, quickly got complicated by DRESS, overlapping with TEN, and the immune system finally got overwhelmed with the fatal HLH. Future clinical studies are needed to identify high-risk cases and the factors that drive their illness trajectory to achieve better patient outcomes.
